# The Role of MLPA in Detecting Syndromic Submicroscopic Copy Number Variations in Normal QF-PCR Miscarriage Specimens

**DOI:** 10.3390/genes16080867

**Published:** 2025-07-24

**Authors:** Gabriela Popescu-Hobeanu, Mihai-Gabriel Cucu, Alexandru Calotă-Dobrescu, Luminița Dragotă, Anca-Lelia Riza, Ioana Streață, Răzvan Mihail Pleșea, Ciprian Laurențiu Pătru, Cristina Maria Comănescu, Ștefania Tudorache, Dominic Iliescu, Florin Burada

**Affiliations:** 1 Doctoral School, University of Medicine and Pharmacy of Craiova, 200349 Craiova, Romania; gmph94@gmail.com; 2 Laboratory of Human Genomics, University of Medicine and Pharmacy of Craiova, 200638 Craiova, Romania; anca.costache@umfcv.ro (A.-L.R.); ioana.streata@umfcv.ro (I.S.); razvan.plesea@umfcv.ro (R.M.P.); florin.burada@umfcv.ro (F.B.); 3 Regional Centre of Medical Genetics Dolj, Emergency Clinical County Hospital Craiova, 200642 Craiova, Romania; alexcalota.crgm@gmail.com (A.C.-D.); luminita.crgm@gmail.com (L.D.); 4 Department of Obstetrics and Gynecology, University of Medicine and Pharmacy of Craiova, 200349 Craiova, Romania; ciprian.patru@umfcv.ro (C.L.P.); stefania.tudorache@gmail.com (Ș.T.); dominic.iliescu@yahoo.com (D.I.); 5 Department of Obstetrics and Gynecology, Emergency Clinical County Hospital, 200642 Craiova, Romania; maria.comanescu@umfcv.ro; 6 Department of Anatomy, University of Medicine and Pharmacy of Craiova, 200349 Craiova, Romania

**Keywords:** pregnancy loss, product of conception, euploidy, chromosome abnormality, CNV, MLPA

## Abstract

**Background/Objectives**: Miscarriage is an increasingly common event worldwide arising from various factors, and identifying its etiology is important for planning and managing any future pregnancies. It is estimated that about half of early pregnancy loss cases are caused by genetic abnormalities, while a significantly lower rate is found in late pregnancy loss. Multiplex ligation-dependent probe amplification (MLPA) can detect small changes within a gene with precise breakpoints at the level of a single exon. The aim of our study was to identify the rate of copy number variations (CNVs) in spontaneous pregnancy loss samples after having previously tested them via quantitative fluorescence PCR (QF-PCR), with no abnormal findings. **Methods**: DNA was extracted from product-of-conception tissue samples, followed by the use of an MLPA kit for the detection of 31 microdeletion/microduplication syndromes (SALSA^®^ MLPA^®^ Probemix P245 Microdeletion Syndromes-1A, MRC-Holland, Amsterdam, The Netherlands). **Results**: A total of 11 (13.1%) out of the 84 successfully tested samples showed CNVs. Duplications accounted for 9.5% of the analyzed samples (eight cases), while heterozygous or hemizygous deletions were present in three cases (3.6%). Among all the detected CNVs, only three were certainly pathogenic (3.6%), with two deletions associated with DiGeorge-2 syndrome and Rett syndrome, respectively, and a 2q23.1 microduplication syndrome, all detected in early pregnancy loss samples. For the remaining cases, additional genetic tests (e.g., aCGH/SNP microarray) are required to establish CNV size and gene content and therefore their pathogenicity. **Conclusions**: MLPA assays seem to have limited value in detecting supplementary chromosomal abnormalities in miscarriages.

## 1. Introduction

There is no general consensus regarding the definition of the term *miscarriage*. However, several global organizations refer to it as pregnancy loss occurring before viability while taking into account factors such as gestation age or fetal weight [1]. The American Society for Reproductive Medicine (ASRM) defines it as the loss of a clinical pregnancy before 20 weeks of gestation [2].

The American College of Obstetricians and Gynecologists (ACOG) defines early pregnancy loss (EPL) as *either an empty gestational sac or a gestational sac containing an embryo or fetus without fetal heart activity* before 13 weeks of gestation [3], and it can be further described as either complete, incomplete, or missed according to clinical and ultrasonographic findings [4]. Taking into account these two definitions, any case of pregnancy loss occurring between 13 and 19 6/7 weeks of gestation will therefore be referred to as late pregnancy loss (LPL) [5].

EPL is an increasingly common event worldwide [6], with 80% of pregnancy loss cases taking place in the first trimester [7]. While previous reports indicate that EPL occurs in approximately 10% of clinically recognized pregnancies [8,9], providing accurate prevalence estimates can prove challenging due to large differences regarding diagnostic methods, demographics, and socio-economic status [10].

Early pregnancy loss has been shown to possess a profound psychological effect on both women and men [11,12], as well as multiple social and economic implications [1,13,14]. Identifying the precise etiology of EPL might help mitigate those issues in addition to playing a vital role in planning and managing any future pregnancies [15].

EPL is a multifactorial condition, arising from various imbalances in embryo/endometrium dynamics [10]. However, around half of early pregnancy loss cases are owed to fetal chromosomal abnormalities—notably aneuploidies [16]. Autosomal aneuploidies are mostly maternally derived [17,18] and are thus strongly linked to advanced maternal age [19].

On the other hand, the causes of late pregnancy loss (LPL) are mostly linked to placental pathology [5,20], factor V and prothrombin mutations [21], the presence of antiphospholipid antibodies [22], or maternal infection [23]. The estimated rate of fetal chromosome abnormalities in LPL is around 15% [24].

Copy number variation (CNV) is a molecular phenomenon describing genome sequence repetition, with the number of repetitions varying between individuals within the same species [25]. CNV length ranges between several dozen base pairs to several megabases and includes deletions, duplications, and complex multisite variants [26,27]. CNVs are deemed *pathogenic* (P) if there are well-documented associations with clinical phenotypes possessing known penetrance and expressivity or if they contain at least one dosage-sensitive region or gene, while *likely pathogenic* CNVs (LP) are variants with ample evidence related to their clinical phenotype, including deletions of the 5′ end or several exons of a haploinsufficient gene or the deletions or duplications associated with them on numerous occasions, with consistent, specific phenotypes [28].

A recent study showed that pCNVs were present in 3.45% of product-of-conception samples [29]. Another large-scale study conducted on EPL samples place the pCNV rate at 2.7% [30].

Multiplex ligation-dependent probe amplification (MLPA) is a technique used for the detection of exon-level CNVs. MLPA probes consist of a pair of oligonucleotides hybridizing with two immediately adjacent target DNA sequences [31]. In case there is a perfect match with their target sequences, the half-probes can be ligated and amplified by using a single pair of fluorescently labeled PCR primers. The resulting PCR products are then separated by capillary electrophoresis, the fluorescence peak being relative to the amount of target DNA in the analyzed sample [32].

MLPA is a high-throughput molecular technique that can detect CNVs in up to 50 different DNA sequences in a single reaction, and it can identify small changes within a gene with precise breakpoints at the level of a single exon [33]. Despite not providing a genome-wide analysis, it is reliable, requires small quantities of DNA, and yields results within two days [34].

The aim of our study was to identify the prevalence of pCNVs in pregnancy loss product-of-conception (POC) samples after having previously tested them via quantitative fluorescence PCR (QF-PCR), with no abnormal findings.

## 2. Materials and Methods

### 2.1. Sample Inclusion

Our study included 91 POC samples showing no abnormal QF-PCR findings using the IVD Devyser Extend kit (Devyser AB, Stockholm, Sweden) that allows for the detection of the aneuploidies of chromosomes 13, 14, 15, 16, 18, 21, 22, X, and Y, as well as triploid and molar pregnancies. All samples were collected and delivered to the Human Genomics Laboratory of the University of Medicine and Pharmacy of Craiova, Romania, for QF-PCR analysis by the Departments of Obstetrics and Gynecology of the Emergency Clinical County Hospital and the Filantropia Clinical Municipal Hospital of Craiova between January 2013 and September 2024. Data regarding reproductive history, including maternal and gestational ages, were recorded. In the present study, maternal ages ranged between 18 and 46 years old, with gestational ages varying between 6 and 19 6/7 weeks. All samples were subsequently split into two separate categories—the early pregnancy loss (EPL) group and the late pregnancy loss (LPL) group.

The study was conducted in accordance with the Declaration of Helsinki and was approved by the Ethics Committee of the University of Medicine and Pharmacy of Craiova, Romania (no. 44/24 March 2022).

### 2.2. Multiplex Ligation-Dependent Probe Amplification (MLPA) Analysis

DNA extraction was performed using Promega Wizard™ Genomic (Promega, Madison, WI, USA), while the SALSA^®^ MLPA^®^ Probemix P245 Microdeletion Syndromes-1A kit (MRC-Holland, Amsterdam, The Netherlands) was used for MLPA analysis according to manufacturer protocols.

The kit contains probes used for the detection of the following syndromes: 1p36 deletion syndrome (1p36), 2p16.1-p15 microdeletion syndrome (2p16.1-p15), 2q23.1 microdeletion/microduplication syndrome (2q23.1), Glass syndrome (2q32-q33), 3q29 microdeletion syndrome (3q29), 3q29 microduplication syndrome (3q29), Wolf–Hirschhorn syndrome (4p16.3), Cri-du-Chat syndrome (5p15), Sotos syndrome (5q35.3), Williams–Beuren syndrome (7q11.23), Williams–Beuren duplication syndrome (7q11.23), Langer–Giedion syndrome (8q24.11-q24.13), 9q22.3 microdeletion syndrome (9q22.3), DiGeorge syndrome-2 (10p14-p13), Prader–Willi syndrome (15q11.2), Angelman syndrome (15q11.2), Witteveen–Kolk/15q24 microdeletion syndrome (15q24), Rubinstein–Taybi syndrome (16p13.3), Miller–Dieker syndrome (17p13.3), lissencephaly-1 (17p13.3), Smith–Magenis syndrome (17p11.2), Potocki–Lupski syndrome (17p11.2), NF1 microdeletion syndrome (17q11.2), Koolen–de Vries syndrome (17q21.31), 17q21.31 microduplication syndrome (17q21.31), DiGeorge syndrome (22q11.21), 22q11.2 microduplication syndrome (22q11.2), distal 22q11.2 deletion syndrome (22q11.2), Phelan–McDermid syndrome (22q13), Rett syndrome (Xq28), and *MECP2* duplication syndrome (Xq28).

The resulting PCR products were migrated on the ABI3730xl platform (Applied Biosystems, Foster City, CA, USA), all resulting data being subsequently analyzed using Coffalyser.Net software v.240129.1959 (MRC-Holland, Amsterdam, The Netherlands).

The MLPA workflow consists of the following steps: DNA denaturation (at 98 °C for 5 min), hybridization (the Hybridization Master Mix was prepared with 1.5 μL of MLPA Buffer and 1.5 μL of MLPA Probemix per reaction and incubated at 95 °C for 1 min, followed by hybridization at 60 °C for 16–20 h), ligation (the Ligation Ligase Master Mix was prepared with 25 μL of ultrapure water, 3 μL of Ligase Buffer A, 3 μL of Ligase Buffer B, and 1 μL of Ligase-65 while keeping the tubes at 54 °C in the thermocycler, adding 32 μL of the Ligase-65 Master Mix and incubating for another 15 min, followed by heat inactivation at 98 °C for 5 min and finally cooling to 20 °C), PCR amplification (the PCR Amplification Polymerase Master Mix was prepared by combining 7.5 μL of ultrapure water, 2 μL of PCR Primer Mix, and 0.5 μL of polymerase per reaction. A total of 10 μL of the Polymerase Master Mix was added to each tube and continued to PCR at 95 °C for 30 s, 60 °C for 30 s, and 72 °C for 60 s for 35 cycles, followed by a final extension at 72 °C for 20 min), post-PCR handling and fragment separation (the PCR products were prepared for capillary electrophoresis with the injection mixture: 0.7 μL of PCR product, 0.3 μL of GeneScan™ 600 LIZ^®^, and 9 μL of HiDi formamide; the plate was sealed, heated at 86 °C for 3 min, and then cooled at 4 °C for 2 min before loading into the ABI3730xl platform), and finally, data analysis using Coffalyser.Net.

SPSS Statistics for Windows, Version 22.0 (IBM SPSS Statistics for Windows, Version 22.0. Armonk, NY, USA: IBM Corp) was used to calculate descriptive statistics.

## 3. Results

MPLA analysis yielded results in 92.3% of cases (84 samples), with seven POC sample results (three EPL and four LPL cases) being deemed inconclusive. Eleven out of the remaining eighty-four samples tested showed CNVs (13.1%). A more detailed view of the results is available in Table 1.

### 3.1. Abnormalities Detected in Early Pregnancy Loss (EPL) Group

**Figure 1:**
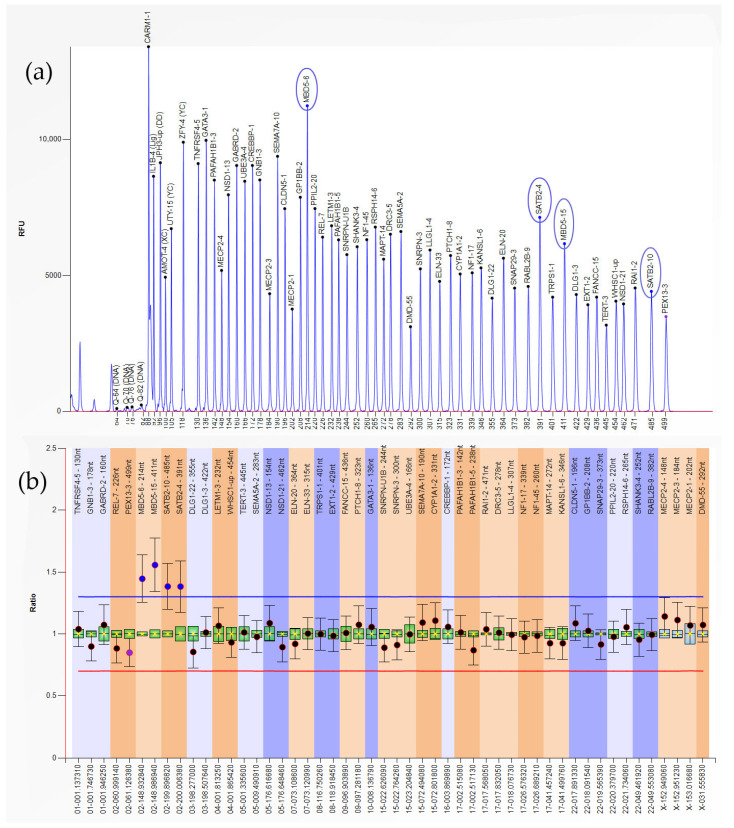
MBD5-6,15; SATB2-4,10 (2q23.1-2q33.1) duplication. (**a**) Plot view of the peak pattern. Abnormal MLPA patterns marked in blue circles. (**b**) MLPA analysis results (normalized sample).

**Figure 2:**
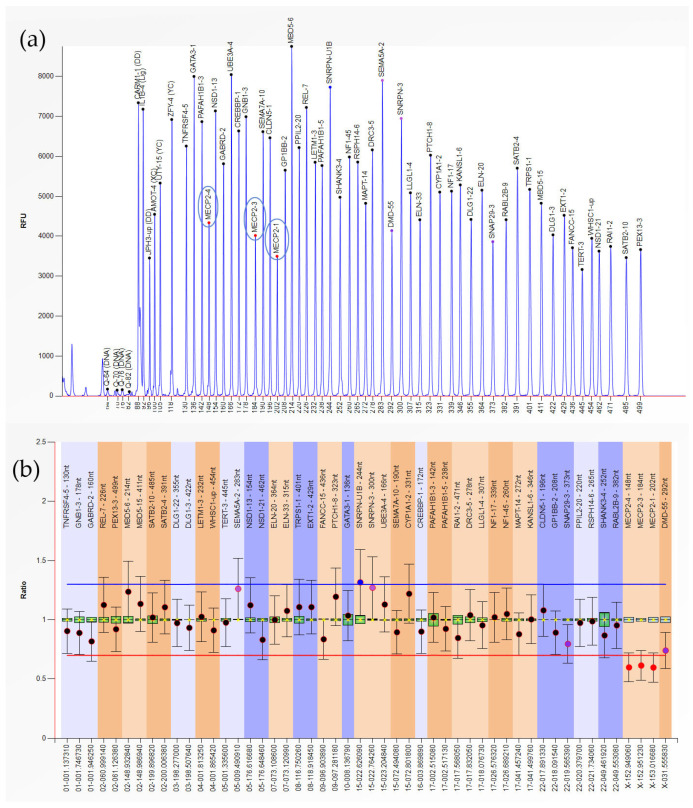
MECP2-1,3,4 deletion. (**a**) Plot view of the peak pattern. Abnormal MLPA patterns marked in blue circles. (**b**) MLPA analysis results (normalized sample).

Out of the 61 samples included in the EPL group, 83.6% had no anomalies detectable by MLPA (*n* = 51), while 10 showed various CNVs (16.4%) (Table 2). We found duplications accounted for 11.5% of the analyzed samples (seven cases) (Figure 1), while heterozygous/hemizygous deletions were present in three cases (4.9%) (Figure 2).

### 3.2. Abnormalities Detected in Late Pregnancy Loss (LPL) Group

Out of the 23 late pregnancy loss POC samples tested, 95.8% of the analyzed specimens (22 cases) showed no chromosome abnormalities. The only CNV detected in the LPL group was a heterozygous duplication of the *CYP1A1* gene, located at 15q24.1 (exon 2).

## 4. Discussion

In our study, we identified eight heterozygous duplications and three deletions. The clinical interpretation of deletions detected by MLPA assays targeting common microdeletion syndromes is relatively straightforward; these disorders possess largely well-defined genotypes and phenotypes [35]. However, interpreting duplications located in the same chromosome regions is challenging, given the fact that the associated phenotypes present either uncertain clinical significance or variable expressivity [34]. It is important to emphasize that MLPA cannot identify the size and gene content of duplications; moreover, the kit we used includes only one probe for many of the chromosomal regions that were investigated.

Three of the eleven detected CNVs are duplications of the *LETM1* and *WHSC1* genes, both located in Wolf–Hirschhorn syndrome (WHS) critical regions [36,37]. While 4p16.3 deletions are consistent with WHS [38], there is some evidence suggesting that the *WHSCR* duplication phenotype presents a certain degree of overlap with the deletion phenotype [39], but there is no evidence showing that small duplications involving *LETM1* and/or *WHSC1* are pCVNs. Both genes are not dosage triplosensitive; despite that, several studies suggest that microscopic *WHSCR* duplications either determine an intermediate phenotype between WHS and trisomy 4p syndrome and therefore represent a different clinical entity altogether [40] or are simply associated with neurodevelopmental disorders [41]; in any case, all duplicated regions spanned more than two genes.

A heterozygous duplication of exon 1 of the *CREBBP* gene was found in an EPL sample. This is a dosage-sensitive gene causing Rubinstein–Taybi syndrome type 1 (RSTS1) when deletions occur with the *CREBBP* gene [42], while there is no evidence for dosage pathogenicity when the gene is duplicated. However, studies have described *CREBBP* gene duplications as central to a separate, clinically recognizable entity, known as 16p13.3 duplication syndrome [43,44,45]. The majority of 16p13.3 duplication syndrome patients present with facial dysmorphism, variable degrees of intellectual disability, growth delay, and anomalies of the brain, heart, genitalia, palate, limbs, and eyes [46].

In addition, we have also found a partial 2q trisomy case, dup(2)(q23.1→q33.1), consistent with distal duplication 2q syndrome [47,48], characterized by developmental delay, intellectual disability, facial dysmorphism, genital anomalies, and clinodactyly [49]. In our case, the exact origin of the duplication must be established, as partial 2q trisomy may occur either *de novo* or as a result of a parental chromosomal rearrangement [48].

On the other hand, we found no discernible pathogenic implications linked to microduplications in the *CYP1A1* gene, an abnormality detected in two POC samples. The probe for the *CYP1A1* gene was included for the detection of Witteveen–Kolk/15q24 microdeletion syndrome caused by the loss of the 15q24 region containing the *CYP1A1* gene. Nevertheless, the duplication of chromosome 15q24 was reported in several patients with developmental delay, dysmorphic features, and finger abnormalities [50,51].

We found deletions in three cases.

A *GATA3* deletion located in the 10p14 region associated with DiGeorge-2 was identified. Microdeletions of chromosome 10p14, affecting, among others, the *GATA3* gene, are defined as DGS/VCFS complex 2 (DiGeorge syndrome/velocardiofacial syndrome complex 2) [52]. In addition to the classic DGS/VCFS phenotype, patients with monosomy 10p also exhibit other clinical features, such as microcephaly, hearing loss, anomalies of the hand, foot, and genitourinary tract, and severe psychomotor impairment [53]. However, phenotypes can vary widely between affected patients. Deletions involving a critical region containing the *GATA3* gene on chromosome 10p lead to hypoparathyroidism, sensorineural deafness, and renal insufficiency syndrome (HDRS) [54,55,56]. HDRS is a rare autosomal dominant disorder caused by the haploinsufficiency of the *GATA3* gene located on chromosome 10p15, which plays an important role in the embryonic development of the central nervous system, inner ear, parathyroid glands, and kidneys [57]. Moreover, due to the location of the DGS2 locus proximal to *GATA3*, distal 10p deletions often result in the presence of both HDRS and DGS [58,59]. There is some evidence that the *GATA3* duplication phenotype might be similar to the deletion phenotype [60], though a general consensus leans towards no evidence for triplosensitivity [61].

A large *MECP2* deletion consistent with Rett syndrome (RTS) was also detected in a male product of conception [62]. Rett syndrome is a severe and progressive neurodevelopmental disorder and the second most prevalent cause of intellectual disability in girls [63,64]. Although Rett syndrome was initially considered to be a dominant X-linked lethal condition in males, point mutations, intragenic, and whole-gene deletions of *MECP2* typically result in severe neonatal encephalopathy [65].

We also found an *RABL2B* deletion classically associated with Phelan–McDermid syndrome (PHMDS), which is linked to global developmental delay, cognitive deficits, and autism spectrum disorder-like behavioral patterns [66]. However, in our case, we found no gains or losses in *SHANK3*, which is located in the minimal critical gene region of PHMDS; nonetheless, we cannot exclude the presence of a small deletion, given the fact that the MLPA kit only contains a single probe targeting exon 4 of *SHANK3*. On the other hand, deletions in the *RABL2B* gene are not expected to contribute to the phenotype of PHMDS, as *RABL2B* is not constrained for protein-truncating variants [67]. In this instance, the interpretation of results proved particularly challenging.

Our overall chromosome abnormality detection rate of 13.1%, but only three were certainly pathogenic (3.3%)—two deletions associated with DiGeorge-2 syndrome and Rett syndrome, respectively, and a 2q23.1 microduplication syndrome, all detected in early pregnancy loss samples. Our detected rate is difficult to compare to other studies due to limited data and marked differences regarding sample inclusion criteria, study reporting, or the type of MLPA assays used; for instance, subtelomeric/subcentromeric probe kits, such as the SALSA^®^ MLPA^®^ Probemix P070 Subtelomeres Mix 2B (MRC-Holland, Amsterdam, The Netherlands), the SALSA^®^ MLPA^®^ Probemix P036-E3 Subtelomeres Mix 1 (MRC-Holland, Amsterdam, The Netherlands) and the SALSA^®^ MLPA^®^ Probemix P181-C1 Centromere mix 1 (MRC-Holland, Amsterdam, The Netherlands). The authors report widely variable results, with the structural abnormality rate generally ranging from 1.5 to 7.8% [68,69,70,71,72,73,74] (Table 3).

Some studies have shown various degrees of association between embryonic or fetal CNVs and pregnancy loss [30,75], with three recurrent pCNVs—7q11.23 microdeletion, 16p13.11 microduplication, and 22q11.2 microdeletion—commonly identified in studies on miscarriage [67,76,77,78]. Other CNVs seem to also be likely reported in the context of early embryonic demise, with future studies required to confirm their role in pregnancy loss [79]. However, there is no definitive proof of CNV causal effects in miscarriages [80].

There are also other molecular techniques available for the evaluation of submicroscopic POC structural gains and losses, with a chromosome microarray (CMA), copy number variation sequencing (CNV-seq), and more recently, next-generation sequencing (NGS) [81].

CMA, which comprises array comparative genomic hybridization (aCGH) and a single-nucleotide polymorphism (SNP) microarray—ideally used together to maximize benefits [82]—is a high-resolution whole-genome molecular technique used to detect CNVs down to 50–100 Kb [83].

A large-scale North American study which included both fresh and formalin-fixed paraffin-embedded product-of-conception samples found a structural abnormality rate of 5.6% when employing CMA as the primary diagnostic method [84]. Another large-scale North American study found deletions or duplications in 6% of cases, including instances of full paternal uniparental disomy (UPD), single UPD, and other complex findings [78].

In a recent meta-analysis performed by Smits et al. [85] and encompassing 55 different studies, the prevalence of chromosomal abnormalities was similar between conventional karyotyping (43–51%) and aCGH (39–57%), with a higher rate of anomalies discernible by an SNP microarray (58–63%). This analysis also showed that the overall structural abnormality rate was 7–9% [85]. Despite currently being the recommended molecular technology for testing POC samples [86,87], CMA is not yet widely available, in addition to being costly.

ACOG guidelines recommend CMA over conventional karyotyping as the preferred method for genetic testing stillbirth (loss at more than 20 week of gestation) but not pregnancy loss [3]. Given the fact that conventional karyotyping analysis is plagued by culture failure and maternal cell contamination but is still able to provide additional cytogenetic information, such as the existence of triploidy and balanced chromosome abnormalities [88], it seems that perhaps a step-by-step, case-by-case approach might yield the best results regarding the etiology of pregnancy loss [89]. Some authors suggest the combined use of QF-PCR and MLPA for the testing of POC samples, followed by CMA in the case of a negative result [90,91]. Other authors suggest using CMA whenever conventional karyotyping fails to yield results, as well as when the normal karyotype is discordant to clinical findings [92].

CNV-seq is a method employing shotgun sequencing that is able to detect CNVs across all 24 chromosomes down to a resolution of 0.1 Mb [93]. A recent study testing miscarriage POC samples found a pCNV prevalence of 6.6% [94], while another study showed a pCNV rate of 4.8% when analyzing spontaneous pregnancy loss samples [95]. On the other hand, other authors showed an overall CNV rate of 13.5%, with only 2.8% being classified as pathogenic or likely pathogenic [30].

Massive parallel or next-generation sequencing (NGS) allows for the generation of data from tens of thousands to billions of templates simultaneously [96], allowing for the detection of variants/mutations in a short amount of time [97]. A large-scale Chinese study consisting of early pregnancy loss POC samples reported 8.2% of pathogenic CNVs when using NGS as a sole method for genetic diagnosis [98].

This study has some limitations. First of all, the small sample size was limited to the analysis of a subgroup of product-of-conception samples. Secondly, the number of probes included in the MLPA kit we used are restricted to target specific genes or regions of interest and additional genetic tests (e.g., array CGH/ SNP microarray) are required to establish CNV size and gene content and therefore their pathogenicity. Thirdly, POC samples underwent QF-PCR testing for aneuploidies of only chromosomes 13, 15, 16, 18, 21, 22, X, and Y. Furthermore, we did not conduct a parental analysis to establish the origin of the detected CNVs in POC.

## 5. Conclusions

MLPA seems to be a less valuable resource for the in-depth analysis of euploid pregnancy losses. Given the limited number of probes included in commercial kits, it can only be used when a recognizable microdeletion or microduplication disorder is suspected or in the case of a positive family history. While some embryonic or fetal CNVs appear to be associated with pregnancy loss, most CNVs cannot be definitively pinpointed as causative, and other underlying causes of miscarriage cannot be ruled out. More accurate are whole-genome techniques such as CMA and their corresponding genome databases, which can provide a better view and interpretation of product-of-conception CNVs, thus providing patients with a correct genetic etiology of pregnancy loss.

## Figures and Tables

**Table 1:** MLPA analysis results for the EPL and LPL groups.

	Results	
	Positive *n*/% *	Negative *n*/% *
Early Pregnancy Loss (EPL)	10 (11.9%)	51 (60.7%)
Late Pregnancy Loss (LPL)	1 (1.2%)	22 (26.2%)
Total *n*/% *	11 (13.1%)	73 (86.9%)

* of all samples analyzed by MLPA, rounded values.

**Table 2:** CNVs detected in EPL samples.

Type of Abnormality	*n*	Gene-Exon (Locus)
Heterozygous Duplication	1	MBD5-6,15; SATB2-4,10 (2q23.1-2q33.1)
	3	LETM1-3; WHSC1-up (4p16.3)
	1	GATA3-1 (10p14)
	1	CYP1A1-2 (15q24.1)
	1	CREBBP-1 (16p13.3)
Heterozygous Deletion	1	GATA3-1 (10p14)
	1	RABL2B-9 (22q13.33)
Hemizygous Deletion	1	MECP2-1,3,4 (Xq28)

**Table 3:** A comparative view of CNV detection rates by MLPA (rounded values).

Study	MLPA Assay	Analyzed Cases (*n*)	Structural Abnormality/CNV (%)
Present study	microdeletion syndromes	84	13.1% (pCNV–3.6%)
Caramins et al., 2011 [70]	subtelomeric	284	2.5%
O’Leary et al., 2013 [69]	unspecified	102	7.8% *
Kim et al., 2014 [74]	subtelomeric	347	3.5%
Zimowski et al., 2016 [73]	subtelomeric/subcentromeric	181	7.2%
Isidori et al., 2017 [72]	subtelomeric	264	1.5%
Lou et al., 2020 [68]	subtelomeric	172	4.1%
Bozhinovski et al., 2024 [71]	subtelomeric	768	3.7%

* might include unspecified cases of monosomy.

## Data Availability

All data presented here are available from the authors upon reasonable request.

## Abbreviations

The following abbreviations are used in this manuscript:

MLPA	Multiplex ligation-dependent probe amplification
CNV	Copy number variation
DNA	Deoxyribonucleic acid
QF-PCR	Quantitative fluorescent-polymerase chain reaction
ASRM	The American Society for Reproductive Medicine
ACOG	The American College of Obstetricians and Gynecologists
EPL	Early pregnancy loss
LPL	Late pregnancy loss
pCNV	Pathogenic copy number variation
lpCNV	Likely pathogenic copy number variation
POC	Product of conception
NF1	Neurofibromatosis type 1
PCR	Polymerase chain reaction
LETM1	Leucine zipper and EF-hand containing transmembrane protein 1
WHS	Wolf–Hirschhorn syndrome
WHSC1	Wolf–Hirschhorn syndrome candidate 1
WHSCR	Wolf–Hirschhorn syndrome critical region
GATA3	GATA binding protein 3
RSTS1	Rubinstein–Taybi syndrome type 1
CYP1A1	Cytochrome P450 family 1 subfamily A member 1
MBD5	Methyl-CpG binding domain protein 5
SATB2	SATB homeobox 2
CREBBP	CREB binding lysine acetyltransferase
RABL2B	RAB, member of RAS oncogene family like 2B
MECP2	Methyl-CpG binding protein 2
HDRS	Hypoparathyroidism, sensorineural deafness, and renal insufficiency syndrome
DGS	DiGeorge syndrome
VCFS	Velocardiofacial syndrome
RTS	Rett syndrome
PHMDS	Phelan–McDermid syndrome
CMA	Chromosomal microarray
CNV-seq	Copy number variation sequencing
aCGH	Array comparative genomic hybridization
SNP	Single-nucleotide polymorphism
UPD	Uniparental disomy
NGS	Next-generation sequencing
